# Aminomaleimide fluorophores: a simple functional group with bright, solvent dependent emission†
†Electronic supplementary information (ESI) available: Experimental protocol, supplementary figures and additional spectroscopic data. See DOI: 10.1039/c5cc02908b



**DOI:** 10.1039/c5cc02908b

**Published:** 2015-05-18

**Authors:** Anne B. Mabire, Mathew P. Robin, Wen-Dong Quan, Helen Willcock, Vasilios G. Stavros, Rachel K. O'Reilly

**Affiliations:** a Department of Chemistry , University of Warwick , Gibbet Hill Road , Coventry , CV4 7AL , UK . Email: r.k.o-reilly@warwick.ac.uk ; Tel: +44 (0)247 652 3236

## Abstract

Amino-substituted maleimides form a new class of highly emissive compounds, with large Stokes shifts (>100 nm) and high quantum yields (up to ∼60%). Emission is responsive to the maleimide's environment with both a red-shift, and quenching, observed in protic polar solvents. Aminomaleimides are easily functionalised, providing a versatile fluorescent probe.

## Abstract 2

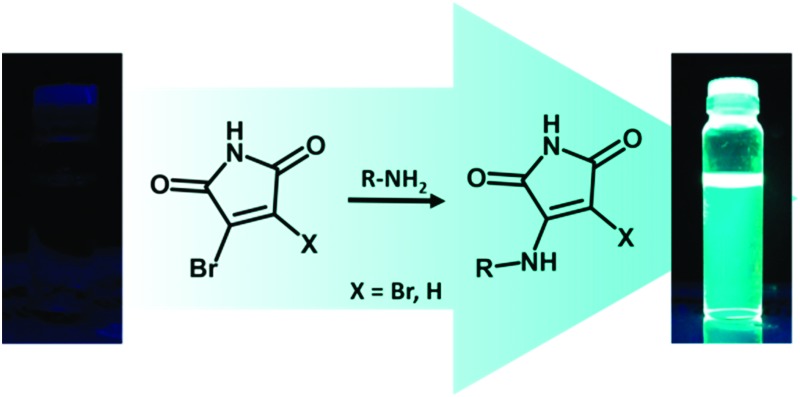
Amino-substituted maleimides form a new class of highly emissive compounds, with large Stokes shifts (>100 nm) and high quantum yields (up to ∼60%).

Fluorophores play an important role as probes in biological systems, for imaging, and for the study of dynamic processes. These applications result from the high sensitivity and ease of measurement for fluorescence emission, and the responsivity of dyes to the physical nature of their environment – for example solvatochromism.^1^ Response of fluorophores to chemical stimuli is also highly desirable, and is frequently utilised in the study of conjugation to biological macromolecules. One mechanism for response is the conversion of a latent quenched fluorophore to an emissive fluorophore upon the desired chemical stimulus. For example the maleimide group is known to be an effective quencher of fluorescence, however when the C=C double bond of the maleimide becomes saturated, *e.g.* upon Michael addition of a thiol, quenching is eliminated and the fluorophore becomes emissive.^2^ Quenching occurs both with direct conjugation of maleimide to fluorophore due to maleimide's low lying nπ* state providing a non-radiative pathway for excited state decay,^3–5^ and also where maleimide and fluorophore are joined by a spacer group due to photoinduced electron transfer (PET) to the C=C double bond.^6–8^ By this process, *N*-fluorophore maleimides have been used as probes for the detection of thiols such as cysteine and glutathione. Bis-maleimides have been used to allow detection of bis-thiols (including reduced disulfides),^9–11^ and maleimide-functional probes have been used for protein labelling,^12^ and both single and two-photon imaging of cells where they allow detection of thiol functional peptides.^9,10,13–16^

Response to chemical stimuli can also be achieved where the reaction of two non-fluorescent species leads to the generation of a fluorophore. For example, the highly efficient tetrazole–alkene/azirine–alkene cycloaddition results in an emissive pyrazoline product.^17^ This cycloaddition reaction has been used to fluorescently label proteins both *in vitro* and within live cells,^18–20^ and for the construction of labelled polymer conjugates with silicon and cellulose surfaces,^21–23^ and with proteins (PEGylation).^24^

We have recently reported an alternative fluorophore generating reaction, whereby the addition of two equivalents of an alkyl thiol to 2,3-dibromomaleimide (DBM) generates the dithiomaleimide (DTM) fluorophore (Scheme 1).^25^ In these compounds rather than quenching emission, the maleimide forms the fluorophore. This OFF-to-ON emission switch upon thiol addition has been used in protein labelling (including disulfide bridging),^25^ polymer and polymer nanoparticle labelling,^25–29^ and the formation of polymer–protein conjugates.^25^ Furthermore, due to the reversibility of thiol addition to DBM,^30^ the alkyl thiols of emissive DTMs can be replaced with aromatic thiols, the result being the formation of a non-emissive DTM. This ON-to-OFF switch has been used as an indicator of polymer nanoparticle morphology transition.^28^

**Scheme 1:**
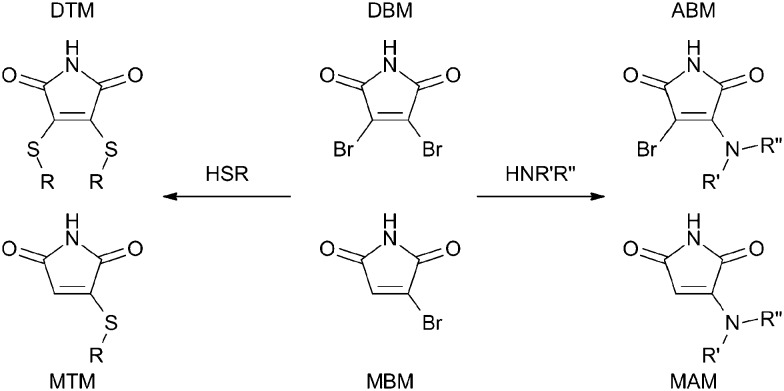
Conversion of mono- or dibromomaleimide (MBM/DBM) to monothio- or dithiomaleimides (MTM/DTM) and monoamino- or aminobromomaleimides (MAM/ABM) by reaction with thiols and amines.

Previous reports have shown that incorporation of the maleimide group into extended π-systems gives a range of dyes with fluorescence quantum yields (*Φ*_f_) of up to 100%, emission maxima ranging from 460–680 nm, and often large Stokes shifts (>100 nm).^31–37^ There are also scattered reports of emissive maleimides with heteroatom substitutions,^38–41^ although a thorough investigation has been lacking. Herein we detail the spectroscopic characterisation of monoaminomaleimides (MAMs), and aminobromomaleimides (ABMs) synthesised by an addition–elimination reaction with monobromomaleimide and 2,3-dibromomaleimide,^42^ in analogy to DTMs (Scheme 1). The ability to form a fluorophore by amino-substitution of a bromomaleimide is of particular interest due to the prevalence of amine groups in biological substrates. We find that MAMs and ABMs are highly emissive with large Stokes shifts (>100 nm) when substituted with alkyl amines, that they show solvent dependent emission wavelength and intensity, and are quenched by the direct conjugation of aromatic rings to the maleimide group.

We began by comparing the fluorescence of these amino-substituted maleimides (MAMs and ABMs) to the previously reported DTM fluorophores (see ESI,† Fig. S1). For dibutylthiomaleimide (**1**),^25^ the polarity of the solvent has an effect on fluorescence, with a decrease in *Φ*_f_ upon increasing the solvent polarity observed. *Φ*_f_ was calculated to be 28% in cyclohexane, 10% in 1,4-dioxane and 0.43% in methanol (see Table 1), relative to the standard quinine sulfate dihydrate.^43^ The molar extinction coefficient (*ε*_max_) varied less significantly with solvent between 4900–5500 M^–1^ cm^–1^ (see ESI†). An increase of the solvent polarity also generated a red-shift in the fluorescence emission maximum, from 486 mm in cyclohexane to 504 mm in 1,4-dioxane and 546 mm in methanol. Interestingly, the equivalent monothiomaleimide (MTM, **2**), synthesised by substitution of monobromomaleimide (MBM) with *n*-butanethiol is non-emissive, having *Φ*_f_ < 0.05% in both 1,4-dioxane and methanol.

In order to compare the emissive properties of the aminomaleimide fluorophores with DTM fluorophores, four model compounds were synthesised and studied (**3–6**, see Table 1), comprising ABMs and MAMs with either secondary butylamine, or tertiary diethylamine substituents. Awuah and Capretta have recently developed a method to synthesise a library of substituted maleimides including amino-substituted maleimides.^42^ They demonstrated that under mild conditions with an excess of amine, only the monoaminated maleimide is formed (with microwave irradiation required to achieve a second substitution). Therefore, as the maleimide undergoes a single amine substitution only, the second substituent of the C=C double bond is retained; bromine or hydrogen depending on the reactant used (DBM or MBM), see Scheme 1. All reactions were performed in THF at room temperature with a small excess of amine, and sodium carbonate as a base. Reactions were completed in 30 min to 2 h. The resultant mixture was washed and purified *via* column chromatography on silica gel. The structures were confirmed by ^1^H NMR spectroscopy, ^13^C NMR spectroscopy, mass spectrometry and infra-red spectroscopy, see ESI.† *Φ*_f_ were determined following the protocol presented by Resch-Genger and co-workers.^43^ For the model compounds **3–6**, measurements were made in cyclohexane, 1,4-dioxane, methanol and water (where solubility allowed) to examine the effect of solvent polarity on emission.

**Table 1:** Structures of substituted maleimides and associated fluorescence quantum yields

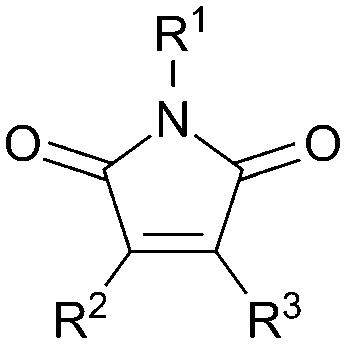				
	R^1^	R^2^	R^3^	*Φ*_f_ ^*a*^/%
**1**	H	S(CH_2_)_3_CH_3_	S(CH_2_)_3_CH_3_	10 (0.43)
**2**	H	H	S(CH_2_)_3_CH_3_	0.043 (0.011)
**3**	H	Br	NH(CH_2_)_3_CH_3_	38 (1.1)
**4**	H	H	NH(CH_2_)_3_CH_3_	59 (2.8)
**5**	H	Br	N(CH_2_CH_3_)_2_	0.15 (0.054)
**6**	H	H	N(CH_2_CH_3_)_2_	0.43 (0.20)
**7**	H	Br	NHCH(CH_3_)_2_	35
**8**	H	Br	NHCH_2_Ph	34
**9**	H	Br	NHPh	0.052
**10**	H	H	NHPh	0.017
**11**	CH_3_	Br	NH(CH_2_)_3_CH_3_	20
**12**	Ph	Br	NH(CH_2_)_3_CH_3_	0.94
**13**	Ph	H	NH(CH_2_)_3_CH_3_	0.085
**14**	Ph	Br	NHPh	0.13

^*a*^Measured in 1,4-dioxane (measured in methanol).

The ABM models **3** (*n*-butylamine substitution) and **5** (diethylamine substitution) presented different fluorescence properties. In 1,4-dioxane, **3** has excitation maxima at 233 nm and 363 nm, with both excitation wavelengths resulting in the same emission maximum at 469 nm, see Fig. 1d and Fig. S2 (ESI†). Stokes shifts for these excitation wavelengths are 236 nm and 106 nm respectively, with this good spectral separation of excitation and emission being important for potential use in FRET experiments.^44^ *Φ*_f_ for **3** was calculated to be 31% in cyclohexane, 38% in 1,4-dioxane, and 1.1% in methanol, see Fig. 1a. The molar extinction coefficient in 1,4-dioxane was found to be 4500 M^–1^ cm^–1^ and the brightness (*Φ*_f_ × *ε*_max_) was calculated to be 1700 M^–1^ cm^–1^. These measurements illustrate that the aminomaleimide fluorophores are significantly brighter than the previously reported DTM fluorophore (**1**). A further advantage for the aminomaleimide (**3**) is that the fluorophore has been generated by single substitution of the maleimide, whereas for thiomaleimides intense emission is only observed for di-substitution.^25^ This property would be particularly beneficial if using a sterically demanding substituent. Interestingly however, ABM synthesised with a secondary amine produced a non-emissive compound (**5**), with *Φ*_f_ < 0.2% in all solvents. This suggests perhaps that the inductive effects of the diethylamine perturbs the electronic structure, as indicated by the red-shift in the absorption maxima going from (**3**) to (**5**) of *ca.* 20 nm (see ESI,† Section II). This, in turn, may introduce a non-radiative pathway for excited state decay that outcompetes fluorescence.

**Fig. 1:**
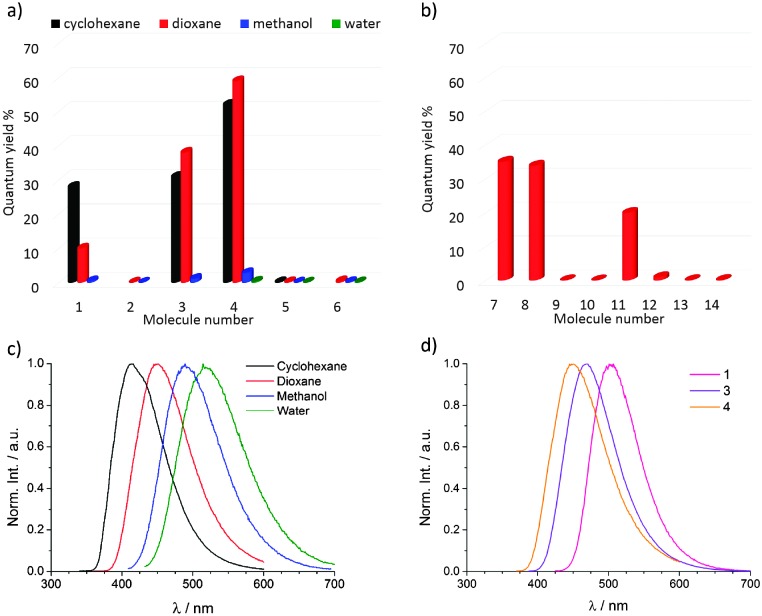
(a) Relative fluorescence quantum yield for the DTM, MTM, ABM and MAM models (**1–6**) in different solvents, (b) relative fluorescence quantum yield of other ABMs and MAMs (**7–14**) in 1,4-dioxane. (c) Emission spectra of **4** in different solvents. (d) Emission spectra of **1**, **3** and **4** in 1,4-dioxane. All spectra were recorded at 10 μM.

The MAM with an *n*-butylamine substituent (**4**) showed very similar excitation and emission spectral profiles to the corresponding ABM (**3**). For example in 1,4-dioxane, **4** displays small spectral shifts in the absorption (233 nm, 346 nm), excitation (236 nm, 346 nm) and emission (450 nm) maxima relative to **3**, see Fig. 1d and Fig. S3 (ESI†). The extinction coefficient for **4** (4900 M^–1^ cm^–1^) is comparable to **3**, while *Φ*_f_, and therefore the brightness, are significantly higher at 59% and 2900 M^–1^ cm^–1^ respectively, see Fig. 1a. As with DTM **1**, the MAM **4** shows a solvatochromic emission (Fig. 1c) with a red-shift upon increasing solvent polarity. However, a significant decrease in *Φ*_f_ was observed in the protic polar solvents methanol and water. A plausible explanation for this could be the formation of an extended hydrogen-bonding network involving the primary amine hydrogen, protic solvent and carbonyl moiety, once again serving to alter the electronic structure and thus facilitate non-radiative decay, effectively switching off fluorescence. Similarly to the non-emissive tertiary ABM **5**, the equivalent MAM (**6**) with a diethylamine substituent displayed *Φ*_f_ < 0.5% in all solvents.

In order to compare the effect of amino-substituents on fluorescence emission, a range of amines were selected for the addition–elimination reaction with 2,3-dibromomaleimide (**7–9**, Table 1) to complement the six model system study. Different primary amines were used; isopropylamine, benzylamine and aniline, with the latter aromatic amine included to illustrate the effect of conjugation of the maleimide with an aromatic ring. All further spectroscopy was performed in 1,4-dioxane as this had given the brightest emission, and a better solubility than cyclohexane. Interestingly, DBM substituted with different primary amines such as isopropylamine (**7**) and benzylamine (**8**) show similar fluorescence properties to **3** (*n*-butylamine substituent) in 1,4-dioxane with absorption maxima at *ca.* 230 nm and *ca.* 363 nm and emission maxima at *ca.* 467 nm (see ESI,† Fig. S4 and S5). *Φ*_f_ for **7** and **8** in 1,4-dioxane were calculated to be, respectively, 35% and 34%, see Table 1 and Fig. 1b. This indicates the versatility of the ABM fluorophores towards further functionalisation through the use of a functional primary amine, as the emission is appears invariant to the groups attached to the primary amine. However, when the maleimide is directly conjugated with a phenyl group (**9**) the product is non-emissive with *Φ*_f_ = 0.05%. This result is consistent with our previous reports of emission quenching where DTM is directly conjugated to aromatic rings.^25,26,28^ A similar result is obtained for MAM with an aniline substituent (**10**), which is also quenched, having *Φ*_f_ = 0.02%.

In addition to the introduction of functionality to these aminomaleimide fluorophores through choice of the amine substituent, further functionality can also be easily incorporated at the maleimide nitrogen.^45,46^ To investigate the effect on emission of *N*-functionalisation of the maleimide, an *N*-methyl ABM with an *n*-butylamine substituent (**11**) was prepared. Incorporation of an alkyl group at the maleimide nitrogen produces a fluorescent compound (**11**), albeit with a reduction in *Φ*_f_ (20%) in comparison with **3** (38%) which is composed of the same R^2^ and R^3^ groups, see Table 1, Fig. 1b and Fig. S6 (ESI†). This does however confirm that *N*-functionalisation is possible, which gives the aminomaleimide fluorophores the versatility to be used as a fluorescent tag through either nitrogen, or as a fluorescent linker group between two species of interest.^27,28^

A phenyl group can also be directly conjugated to the maleimide through the maleimide nitrogen (R^1^), however this has the same effect on fluorescence intensity as conjugation through the amine (R^3^). Namely, fluorescence quenching was observed for the molecules **12**, **13** and **14**, see Fig. 1b.

A MAM with phenyl substituents on both nitrogens (R^1^ = R^3^ = Ph) has recently been reported as an aggregation induced emission (AIE) fluorophore.^47,48^ Our related compounds substituted with one phenyl group (**9**, **10**, **12** and **13**) were tested and found not to display AIE, behaving as self-quenching fluorophores in 1,4-dioxane:water mixtures (see ESI,† Fig. S7–S11). Interestingly the equivalent ABM with R^1^ = R^3^ = Ph (**14**) was also found to not exhibit AIE, which may be the result of the Br substituent inhibiting effective aggregation.

In conclusion, we have presented a library of aminomaleimides (MAMs and ABMs), which show an intense fluorescence emission if the maleimide is substituted with alkyl primary amines. The quenching of the fluorescence by conjugation of the maleimide with a phenyl group was confirmed *via* insertion of the phenyl group at different sites, while substitution with a secondary amine also yields a non-emissive product. The study of the effect of solvent on the emission wavelength and fluorescence quantum yield showed a dependence on the solvent polarity, with a significant (100 nm) red-shift of emission maxima observed, and the highest fluorescence quantum yields in aprotic solvents. With the design rules for aminomaleimide fluorophores established herein, we believe that the combination of ease of synthesis, versatile functionalisation, high fluorescence quantum yields, large Stokes shifts and solvent dependent emission make these aminomaleimide dyes an important tool for fluorescent labelling. Computation studies are currently underway to provide deeper insight into these design rules, specifically how electronic structure, and thus excited state dynamics, is influenced by functionalisation and solvent polarity.

The authors thank Dr Tolga Karsili (T. U. Munich) for helpful discussions, and BP, the IAS at the University of Warwick, the ERC (Grant No. 615142) and the EPSRC for funding.

## References

[cit1] Lakowicz J. R., Principles of Fluorescence Spectroscopy, Springer, 2009.

[cit2] Chen X., Zhou Y., Peng X., Yoon J. (2010). Chem. Soc. Rev..

[cit3] Langmuir M. E., Yang J.-R., Moussa A. M., Laura R., LeCompte K. A. (1995). Tetrahedron Lett..

[cit4] Kand D., Kalle A. M., Talukdar P. (2013). Org. Biomol. Chem..

[cit5] Sippel T. O. (1981). J. Histochem. Cytochem..

[cit6] Prasanna de Silva A., Nimal Gunaratne H. Q., Gunnlaugsson T. (1998). Tetrahedron Lett..

[cit7] Guy J., Caron K., Dufresne S., Michnick S. W., Skene W. G., Keillor J. W. (2007). J. Am. Chem. Soc..

[cit8] Youziel J., Akhbar A. R., Aziz Q., Smith M. E. B., Caddick S., Tinker A., Baker J. R. (2014). Org. Biomol. Chem..

[cit9] Chen Y., Clouthier C. M., Tsao K., Strmiskova M., Lachance H., Keillor J. W. (2014). Angew. Chem., Int. Ed..

[cit10] Pan X., Liang Z., Li J., Wang S., Kong F., Xu K., Tang B. (2015). Chem. – Eur. J..

[cit11] Gao F., Chen H., Xu S., Cheng Y., Ma Y. (2013). Talanta.

[cit12] Wache N., Scholten A., Klüner T., Koch K.-W., Christoffers J. (2012). Eur. J. Org. Chem..

[cit13] Liu Y., Liu Y., Liu W., Liang S. (2015). Spectrochim. Acta, Part A.

[cit14] Zhang Y., Huo F., Yin C., Yue Y., Hao J., Chao J., Liu D. (2015). Sens. Actuators, B.

[cit15] Yang Y., Huo F., Yin C., Chao J., Zhang Y. (2015). Dyes Pigm..

[cit16] Guo X., Zhang X., Wang S., Li S., Hu R., Li Y., Yang G. (2015). Anal. Chim. Acta.

[cit17] Lim R. K. V., Lin Q. (2011). Acc. Chem. Res..

[cit18] Song W., Wang Y., Qu J., Madden M. M., Lin Q. (2008). Angew. Chem., Int. Ed..

[cit19] Song W., Wang Y., Qu J., Lin Q. (2008). J. Am. Chem. Soc..

[cit20] Song W., Wang Y., Yu Z., Vera C. I. R., Qu J., Lin Q. (2010). ACS Chem. Biol..

[cit21] Dietrich M., Delaittre G., Blinco J. P., Inglis A. J., Bruns M., Barner-Kowollik C. (2012). Adv. Funct. Mater..

[cit22] Tischer T., Rodriguez-Emmenegger C., Trouillet V., Welle A., Schueler V., Mueller J. O., Goldmann A. S., Brynda E., Barner-Kowollik C. (2014). Adv. Mater..

[cit23] Hufendiek A., Barner-Kowollik C., Meier M. A. R. (2015). Polym. Chem..

[cit24] Lim R. K. V., Lin Q. (2010). Chem. Commun..

[cit25] Robin M. P., Wilson P., Mabire A. B., Kiviaho J. K., Raymond J. E., Haddleton D. M., O'Reilly R. K. (2013). J. Am. Chem. Soc..

[cit26] Robin M. P., O'Reilly R. K. (2014). Chem. Sci..

[cit27] Robin M. P., Mabire A. B., Damborsky J. C., Thom E. S., Winzer-Serhan U. H., Raymond J. E., O'Reilly R. K. (2013). J. Am. Chem. Soc..

[cit28] Mabire A. B., Robin M. P., Willcock H., Pitto-Barry A., Kirby N., O'Reilly R. K. (2014). Chem. Commun..

[cit29] Robin M. P., Raymond J. E., O'Reilly R. K. (2015). Mater. Horiz..

[cit30] Smith M. E. B., Schumacher F. F., Ryan C. P., Tedaldi L. M., Papaioannou D., Waksman G., Caddick S., Baker J. R. (2010). J. Am. Chem. Soc..

[cit31] Wu W. C., Yeh H. C., Chan L. H., Chen C. T. (2002). Adv. Mater..

[cit32] Chiu C.-W., Chow T. J., Chuen C.-H., Lin H.-M., Tao Y.-T. (2003). Chem. Mater..

[cit33] Yeh H.-C., Wu W.-C., Chen C.-T. (2003). Chem. Commun..

[cit34] Xie H.-d., Ho L., Truelove M., Corry B., Stewart S. (2010). J. Fluoresc..

[cit35] Onimura K., Matsushima M., Nakamura M., Tominaga T., Yamabuki K., Oishi T. (2011). J. Polym. Sci., Part A: Polym. Chem..

[cit36] Nacheva K. P., Maza W. A., Myers D. Z., Fronczek F. R., Larsen R. W., Manetsch R. (2012). Org. Biomol. Chem..

[cit37] Lauer M. H., Drekener R. L., Correia C. R. D., Gehlen M. H. (2014). Photochem. Photobiol. Sci..

[cit38] Leismann H., Marzolph G., Scharf H.-D., Behruzi M. (1983). Chem. Ber..

[cit39] Bodige S. G., Méndez-Rojas M. A., Watson W. H. (1999). J. Chem. Crystallogr..

[cit40] Smith T. L., US Pat., 4680272, 1987.

[cit41] Cui J., Wang S., Huang K., Li Y., Zhao W., Shi J., Gu J. (2014). New J. Chem..

[cit42] Awuah E., Capretta A. (2011). J. Org. Chem..

[cit43] Würth C., Grabolle M., Pauli J., Spieles M., Resch-Genger U. (2013). Nat. Protoc..

[cit44] Kim S. H., Gunther J. R., Katzenellenbogen J. A. (2008). Org. Lett..

[cit45] Walker M. A. (1995). J. Org. Chem..

[cit46] Castañeda L., Wright Z. V. F., Marculescu C., Tran T. M., Chudasama V., Maruani A., Hull E. A., Nunes J. P. M., Fitzmaurice R. J., Smith M. E. B., Jones L. H., Caddick S., Baker J. R. (2013). Tetrahedron Lett..

[cit47] Kato T., Naka K. (2012). Chem. Lett..

[cit48] Boominathan M., Sathish V., Nagaraj M., Bhuvanesh N., Muthusubramanian S., Rajagopal S. (2013). RSC Adv..

