# Supplementary material for: Aminomaleimide fluorophores: a simple functional group with bright, solvent dependent emission
Source: Chem Commun (Camb). 2015 May 18;51(47):9733–6. doi: 10.1039/c5cc02908b (PMC4540011; doi:10.1039/c5cc02908b)
Supplement: Supplementary file 1 [file CC-051-C5CC02908B-s001.pdf]

Electronic Supplementary Material (ESI) for Chemical Communications.
This journal is © The Royal Society of Chemistry 2015

Electronic Supplementary Information

for

Aminomaleimide fluorophores: a simple functional group with bright, solvent dependent emission

Anne B. Mabire,‡[a] Mathew P. Robin,‡[a] Wen-Dong Quan,[a] Helen Willcock,[a] Vasilios G. Stavros,[a] and Rachel K. O'Reilly[a]*

[a]Department of Chemistry, University of Warwick, Coventry, Gibbet Hill, CV4 7AL, U.K.

E-mail: r.k.o-reilly@warwick.ac.uk; Tel: + 44 (0)247 652 3236

Contents

I. Experimental ... 3

1. Materials ... 3
2. Apparatus ... 3
3. General procedure for the synthesis of aminobromomaleimides ... 4

3-Bromo-4-(butylamino)-2,5-dihydro-1H-pyrrole-2,5-dione (3) ... 4

3-Bromo-4-(diethylamino)-2,5-dihydro-1H-pyrrole-2,5-dione (5) ... 4

3-Bromo-4-(isopropylamino)-2,5-dihydro-1H-pyrrole-2,5-dione (7) ... 5

3-Bromo-4-(benzylamino)-2,5-dihydro-1H-pyrrole-2,5-dione (8) ... 5

3-Bromo-4-(phenylamino)-2,5-dihydro-1H-pyrrole-2,5-dione (9) ... 6

3-Bromo-4-(butylamino)-1-methyl-2,5-dihydro-1H-pyrrole-2,5-dione (11) ... 6

3-Bromo-4-(butylamino)-1-phenyl-2,5-dihydro-1H-pyrrole-2,5-dione (12) ... 7

3-Bromo-1-phenyl-4-(phenylamino)-1H-pyrrole-2,5-dione (14) ... 7

4. General procedure for the synthesis of monoaminomaleimides ... 8

3-(butylamino)-2,5-dihydro-1H-pyrrole-2,5-dione (4) ... 8

3-(diethylamino)-2,5-dihydro-1H-pyrrole-2,5-dione (6) ... 8

3-(phenylamino)-2,5-dihydro-1H-pyrrole-2,5-dione (10) ... 9

3-(butylamino)-1-phenyl-2,5-dihydro-1H-pyrrole-2,5-dione (13) ... 9

5. Synthesis of 3-(butylsulfanyl)-2,5-dihydro-1H-pyrrole-2,5-dione (2) ... 10

II. Fluorophore characterisation ... 11

III. Supplementary figures ... 12

IV. References ... 18

I. Experimental

1. Materials

Spectroscopy grade 1,4-dioxane, cyclohexane, and methanol were purchased from VWR. Water for spectroscopy was purified to a resistivity of 18.2 MΩ·cm using a Millipore Simplicity Ultrapure water system. 3,4-Dibromo-1-phenyl-2,5-dihydro-1H-pyrrole-2,5-dione was synthesised according to the procedure of Muus *et al.*[1] 3-Bromo-2,5-dihydro-1H-pyrrole-2,5-dione (monobromomaleimide) was synthesised according to the procedure of Vanel *et al.*[2] 3-Bromo-1-phenyl-2,5-dihydro-1H-pyrrole-2,5-dione was synthesised according to the procedure of Martinez-Ariza *et al.*[3] 2,3-Dibutylthiomaleimide (**1**) was synthesised as previously reported.[4] All other chemicals were purchased from Aldrich, Fluka or Acros and used as received.

2. Apparatus

^{1}H and ^{13}C NMR spectra were recorded on a Bruker DPX-400, or AV500 spectrometer at room temperature unless otherwise stated. Chemical shifts are given in ppm downfield from the internal standard tetramethylsilane (TMS). Infrared spectra were recorded (neat) on a PerkinElmer, Spectrum 100 FT-IR Spectrometer. High Resolution Mass Spectrometry (HR-MS) was conducted on a Bruker UHR-Q-ToF MaXis with electrospray ionisation. Fluorescence spectra were recorded using an Agilent Cary Eclipse Fluorescence spectrophotometer. UV-vis spectroscopy was carried out on a Perkin Elmer Lambda 35 UV/vis spectrometer or an Agilent Cary 60 UV-Vis Spectrophotometer. Quartz cells with screw caps and four polished sides (Starna) were used for fluorescence and UV-vis measurements.

3. General procedure for the synthesis of aminobromomaleimides

Reactions were performed according to the protocol established by Awuah and Capretta.[5] All reactions were performed in THF (20 mL) at room temperature with 2,3-dibromomaleimide (1 eq.), sodium carbonate (2.5 eq.) and a small excess of amine (1.05-1.1 eq.). Consumption of 2,3-dibromomaleimide was monitored by TLC, and was complete within 30 min to 2 h. The solvent was then evaporated under reduced pressure and the residue was taken up with 150 mL of DCM. The resultant mixture was washed with water (2 × 150 mL), dried with magnesium sulfate and purified *via* column chromatography on silica gel with petroleum ether/ethyl acetate.

3-Bromo-4-(butylamino)-2,5-dihydro-1H-pyrrole-2,5-dione (3)

In this case, 2,3-dibromomaleimide (500 mg, 1.96 mmol) and *n*-butylamine (151 mg, 2.06 mmol) were used in the general procedure described above. The product was obtained as a yellow powder (274 mg, 1.11 mmol, 57%) after purification by column chromatography on silica gel using a mixture of 5:1, petroleum ether and ethyl acetate. ^{1}H NMR ($CDCl_3$, 500 MHz, ppm) δ = 7.75 (br, 1H), 5.51 (br, 1H), 3.65 (q, ^3J = 8 Hz, 2H), 1.65 (quin, ^3J = 8 Hz, 2H), 1.42 (sex, ^3J = 8 Hz, 2H), 0.97 (t, ^3J = 8 Hz, 3H); ^{13}C NMR ($CDCl_3$, 125 MHz, ppm) δ = 167.6, 165.8, 143.7, 69.6, 42.7, 35.6, 19.6, 13.7; FTIR (neat) ν_{max} / cm^{-1} 3336 (H-N of amine), 3145 (H-N of maleimide) 1761 and 1708 (C=O of maleimide), 1630 (C=C of maleimide); HR-MS (MaXis) m/z found 268.9892, calc. 268.9896 ($[C_8H_{11}BrN_2O_2+Na]^+$, 100%).

3-Bromo-4-(diethylamino)-2,5-dihydro-1H-pyrrole-2,5-dione (5)

In this case, 2,3-dibromomaleimide (3.00 g, 11.8 mmol) and diethylamine (947 mg, 12.9 mmol) were used in the general procedure described above. The product was obtained as

a yellow powder (2.11 g, 8.55 mmol, 72%) after purification by column chromatography on silica gel using a gradient from 6:1 to 4:1 of petroleum ether and ethyl acetate. ^{1}H NMR ($CDCl_3$, 400 MHz, ppm) δ = 7.56 (br, 1H), 3.76 (q, ^3J = 7 Hz, 4H), 1.28 (t, ^3J = 7 Hz, 6H); ^{13}C NMR ($CDCl_3$, 100 MHz, ppm) δ = 166.6, 165.5, 144.0, 78.2, 45.8, 14.6; FTIR (neat) ν_{max} / cm^{-1} 3228 (H-N of maleimide) 1756 and 1707 (C=O of maleimide), 1616 (C=C of maleimide); HR-MS (MaXis) m/z found 268.9895, calc. 268.9896 ($[C_8H_{11}BrN_2O_2+Na]^+$, 100%).

3-Bromo-4-(isopropylamino)-2,5-dihydro-1H-pyrrole-2,5-dione (7)

In this case, 2,3-dibromomaleimide (500 mg, 1.96 mmol) and isopropylamine (122 mg, 2.06 mmol) were used in the general procedure described above. The product was obtained as an orange powder (176 mg, 0.76 mmol, 39%) after purification by column chromatography on silica gel using a mixture of 5:1, petroleum ether and ethyl acetate. ^{1}H NMR ($CDCl_3$, 500 MHz, ppm) δ = 7.86 (br, 1H), 5.33 (br, 1H), 4.41 (m, 1H), 1.30 (d, ^3J = 5 Hz, 6H); ^{13}C NMR ($CDCl_3$, 125 MHz, ppm) δ = 167.8, 166.0, 142.8, 69.6, 44.7, 23.6; FTIR (neat) ν_{max} / cm^{-1} 3322 (H-N amine), 3189 (H-N of maleimide), 1764 and 1708 (C=O of maleimide), 1639 (C=C of maleimide); HR-MS (MaXis) m/z found 254.9738, calc. 254.9740 ($[C_7H_9BrN_2O_2+Na]^+$, 100%).

3-Bromo-4-(benzylamino)-2,5-dihydro-1H-pyrrole-2,5-dione (8)

In this case, 2,3-dibromomaleimide (500 mg, 1.96 mmol) and benzylamine (221 mg, 2.06 mmol) were used in the general procedure described above. The product was obtained as an orange powder (61 mg, 0.22 mmol, 11%) after purification by column chromatography on silica gel using a mixture of 5:1, petroleum ether and ethyl acetate. ^{1}H NMR ($CDCl_3$, 400 MHz, ppm) δ = 7.36 (m, 5H), 7.11 (br, 1H), 5.60 (br, 1H), 4.86 (d, ^3J = 8 Hz, 2H); ^{13}C

NMR (CD_3OD, 125 MHz, ppm) 169.3, 166.5, 157.3, 138.7, 128.3, 127.1, 126.7, 103.22, 45.2; FTIR (neat) ν_{max} / cm^{-1} 3325 (H-N of amine), 3157 (H-N of maleimide) 1772 and 1714 (C=O of maleimide), 1645 (C=C of maleimide); HR-MS (MaXis) m/z found 302.9749, calc. 302.9740 ($[C_{11}H_9BrN_2O_2+Na]^+$, 100%).

3-Bromo-4-(phenylamino)-2,5-dihydro-1H-pyrrole-2,5-dione (9)

In this case, 2,3-dibromomaleimide (500 mg, 1.96 mmol) and aniline (192 mg, 2.06 mmol) were used in the general procedure described above. The product was obtained as an orange powder (103 mg, 0.39 mmol, 19%) after purification by column chromatography on silica gel using a mixture of 5:1, petroleum ether and ethyl acetate. ^1H NMR (CD_3OD, 500 MHz, ppm) δ = 7.38 (m, 4H), 7.23 (m, 6H); ^{13}C NMR (CD_3OD, 125 MHz, ppm) 169.3, 166.9, 141.7, 136.3, 128.0, 125.4, 124.6, 79.9; FTIR (neat) ν_{max} / cm^{-1} 3303 (H-N of amine), 3188 (H-N of maleimide) 1766 and 1706 (C=O of maleimide), 1636 (C=C of maleimide); HR-MS (MaXis) m/z found 288.9596, calc. 288.9583 ($[C_{10}H_7BrN_2O_2+Na]^+$, 100%).

3-Bromo-4-(butylamino)-1-methyl-2,5-dihydro-1H-pyrrole-2,5-dione (11)

In this case, 2,3-dibromo-*N*-methylmaleimide (750 mg, 2.79 mmol) and *n*-butylamine (214 mg, 2.93 mmol) were used in the general procedure described above. The product was obtained as a yellow-orange powder (448 mg, 1.72 mmol, 62%) after purification by column chromatography on silica gel using a mixture of 5:1, petroleum ether and ethyl acetate. ^1H NMR ($CDCl_3$, 500 MHz, ppm) δ = 5.46 (br, 1H), 3.63 (q, ^3J = 8 Hz, 2H), 3.02 (s, 3H), 1.63 (quin, ^3J = 8 Hz, 2H), 1.42 (sex, ^3J = 8 Hz, 2H), 0.97 (t, ^3J = 8 Hz, 3H); ^{13}C NMR ($CDCl_3$, 125 MHz, ppm) δ = 168.1, 166.2, 143.3, 69.6, 42.8, 32.7, 24.4, 19.6, 13.7; FTIR (neat) ν_{max} / cm^{-1} 3342 (H-N of amine), 1769 and 1709 (C=O of maleimide), 1647 (C=C of maleimide); HR-MS (MaXis) m/z found 283.0048, calc. 283.0053 ($[C_9H_{13}BrN_2O_2+Na]^+$, 100%).

3-Bromo-4-(butylamino)-1-phenyl-2,5-dihydro-1H-pyrrole-2,5-dione (12)

In this case, 3,4-dibromo-1-phenyl-2,5-dihydro-1H-pyrrole-2,5-dione (150 mg, 0.46 mmol) and *n*-butylamine (35 mg, 0.48 mmol) were used in the general procedure described above. The product was obtained as a yellow-orange powder (26 mg, 0.08 mmol, 25%) after purification by column chromatography on silica gel using a mixture of 5:1, petroleum ether and ethyl acetate. ^{1}H NMR ($CDCl_3$, 400 MHz, ppm) δ = 7.44 (m, 2H), 7.34 (m, 3H), 5.57 (br, 1H), 3.70 (q, ^3J = 8 Hz, 2H), 1.69 (quin, ^3J = 8 Hz, 2H), 1.45 (sex, ^3J = 8 Hz, 2H), 0.99 (t, ^3J = 8 Hz, 3H);); ^{13}C NMR (CD_3OD, 125 MHz, ppm) δ = 167.4, 164.5, 144.2, 132.0, 131.6, 128.5, 127.4, 127.2, 126.0, 120.4, 42.1, 32.9, 19.3, 12.8; FTIR (neat) ν_{max} / cm^{-1} 3321 (H-N of amine), 1770 and 1712 (C=O of maleimide), 1649 (C=C of maleimide); HR-MS (MaXis) m/z found 345.0207, calc. 345.0209 ($[C_{14}H_{15}BrN_2O_2+Na]^+$, 100%).

3-Bromo-1-phenyl-4-(phenylamino)-1H-pyrrole-2,5-dione (14)

In this case, 3,4-dibromo-1-phenyl-2,5-dihydro-1H-pyrrole-2,5-dione (150 mg, 0.46 mmol) and aniline (45 mg, 0.48 mmol) were used in the general procedure described above. The product was obtained as a yellow powder (66 mg, 0.19 mmol, 42%) after purification by column chromatography on silica gel using a mixture of 5:1, petroleum ether and ethyl acetate. ^{1}H NMR (CD_3OD, 400 MHz, ppm) δ = 7.47 (m, 2H), 7.40 (m, 5H), 7.28 (m, 3H); ^{13}C NMR ($CDCl_3$, 125 MHz, ppm) δ = 166.6, 165.6, 139.9, 135.1, 131.5, 129.2, 128.8, 127.9, 126.6, 125.8, 124.7, 81.2; FTIR (neat) ν_{max} / cm^{-1} 3309 (H-N of amine), 1769 and 1706 (C=O of maleimide), 1647 (C=C of maleimide); HR-MS (MaXis) m/z found 364.9905, calc. 364.9896 ($[C_{16}H_{11}BrN_2O_2+Na]^+$, 100%).

4. General procedure for the synthesis of monoaminomaleimides

All reactions were performed in THF (20 mL) at room temperature with bromomaleimide (1 eq.), sodium carbonate (2.5 eq.) and a small excess of amine (1.05-1.1 eq.). Consumption of bromomaleimide was monitored by TLC and was complete within 30 min to 2 h. The reaction mixture was filtered, the filtrate collected and the solvent removed under reduced pressure. The resultant residue was then purified *via* column chromatography on silica gel with petroleum ether/ethyl acetate.

3-(butylamino)-2,5-dihydro-1H-pyrrole-2,5-dione (4)

In this case, monobromomaleimide (500 mg, 2.84 mmol) and *n*-butylamine (218 mg, 2.98 mmol) were used in the general procedure described above. The product was obtained as a yellow powder (354 mg, 2.11 mmol, 74%) after purification by column chromatography on silica gel using a mixture of 5:1, petroleum ether and ethyl acetate. ^{1}H NMR ($CDCl_3$, 400 MHz, ppm) δ = 7.07 (br, 1H), 5.37 (br, 1H), 4.81 (s, 1H), 3.18 (q, ^3J = 7 Hz, 2H), 1.63 (m, 2H), 1.41 (m, 2H), 0.96 (t, ^3J = 7 Hz); ^{13}C NMR ($CDCl_3$, 100 MHz, ppm) δ = 172.7, 167.9, 149.9, 85.0, 44.1, 30.5, 20.0, 13.7; FTIR (neat) ν_{max} / cm^{-1} 3311 (H-N of amine), 3187 (H-N of maleimide), 1760 and 1702 (C=O of maleimide), 1624 (C=C of maleimide); HR-MS (MaXis) m/z found 191.0789, calc. 191.0791 ($[C_8H_{12}N_2O_2+Na]^+$, 100%).

3-(diethylamino)-2,5-dihydro-1H-pyrrole-2,5-dione (6)

In this case, monobromomaleimide (500 mg, 2.84 mmol) and diethylamine (218 mg, 2.98 mmol) were used in the general procedure described above. The product was obtained as a yellow powder (375 mg, 2.23 mmol, 79%) after purification by column chromatography on silica gel using a mixture of 3:1, petroleum ether and ethyl acetate. ^{1}H NMR ($CDCl_3$, 500 MHz, 248 K, ppm) δ = 7.68 (br, 1H), 4.80 (d, ^5J = 2 Hz, 1H), 3.84 (q, ^3J = 7 Hz, 2H),

3.26 (q, ^3J = 7 Hz, 2H), 1.23 (t, ^3J = 7 Hz, 6H); ^{13}C NMR ($CDCl_3$, 125 MHz, 248 K, ppm) δ = 172.3, 167.5, 149.6, 86.8, 47.4, 44.4, 14.8, 11.0; FTIR (neat) ν_{max} / cm^{-1} 3111 (H-N of maleimide), 1750 and 1681 (C=O of maleimide), 1602 (C=C of maleimide); HR-MS (MaXis) m/z found 191.0790, calc. 191.0791 ($[C_8H_{12}N_2O_2+Na]^+$, 100%).

3-(phenylamino)-2,5-dihydro-1H-pyrrole-2,5-dione (10)

This compound was synthesised according to the procedure of Bowler *et al.*[6]

^{1}H NMR ($CDCl_3$, 400 MHz, ppm) δ = 7.1 (br m, 5H), 5.53 (s, 1H); ^{13}C NMR (CD_3OD, 100 MHz, ppm) δ = 176.4, 170.4, 145.9, 140.8, 130.5, 125.3, 120.6, 90.0; FTIR (neat) ν_{max} / cm^{-1} 3251 (H-N of aniline), 1769 and 1688 (C=O of maleimide), 1619 (C=C of maleimide); HR-MS (MaXis) m/z found 211.0478, calc. 211.0476 ($[C_{10}H_8N_2O_2+Na]^+$, 100%).

3-(butylamino)-1-phenyl-2,5-dihydro-1H-pyrrole-2,5-dione (13)

In this case, 3-bromo-1-phenyl-2,5-dihydro-1H-pyrrole-2,5-dione (400 mg, 1.59 mmol) and *n*-butylamine (232 mg, 3.17 mmol) were used in the general procedure described above. The product was obtained as a yellow-green powder (258 mg, 1.06 mmol, 66%) after purification by column chromatography on silica gel using a mixture of 5:1, petroleum ether and ethyl acetate. ^{1}H NMR ($CDCl_3$, 400 MHz, ppm) δ = 7.44 (t, ^3J = 8 Hz, 2H), 7.36 (m, 2H), 7.32 (t, ^3J = 7 Hz, 1H), 5.50 (br, 1H), 4.97 (s, 1H), 3.24 (q, ^3J = 7 Hz, 2H), 1.67 (m, 2H), 1.44 (m, 2H), 0.98 (t, ^3J = 7 Hz, 3H); ^{13}C NMR ($CDCl_3$, 100 MHz, ppm) δ = 171.1, 166.3, 149.1, 131.9, 128.9, 127.2, 125.8, 84.1, 44.1, 30.1, 20.0, 13.6; FTIR (neat) ν_{max} / cm^{-1} 3253 (H-N of amine), 1763 and 1698 (C=O of maleimide), 1648 (C=C of maleimide); HR-MS (MaXis) m/z found 267.1105, calc. 267.1104 ($[C_{14}H_{16}N_2O_2+Na]^+$, 100%).

5. Synthesis of 3-(butylsulfanyl)-2,5-dihydro-1H-pyrrole-2,5-dione (2)

To a solution of monobromomaleimide (400 mg, 2.27 mmol) in methanol (8 ml) was added dropwise a solution of sodium acetate (186 mg, 2.27 mmol) and *n*-butanethiol (204 mg, 2.27 mmol) in methanol (8 ml). After 2 h the solvent was removed under reduced pressure, and the resultant residue dissolved in ethyl acetate and filtered and the filtrate concentrated under reduced pressure. The product was obtained as a yellow powder (317 mg, 1.71 mmol, 75%) after purification by column chromatography on silica gel using a mixture of 4:1, petroleum ether and ethyl acetate. ^{1}H NMR ($CDCl_3$, 400 MHz, ppm) δ = 7.34 (br, 1H), 6.05 (s, 1H), 2.92 (t, ^{3}J = 7 Hz), 1.75 (m, 2H), 1.49 (m, 2H), 0.97 (t, ^{3}J = 7 Hz); ^{13}C NMR (CD_3OD, 100 MHz, ppm) δ = 168.8, 167.3, 152.0, 117.5, 31.0, 29.0, 21.4, 12.9; FTIR (neat) ν_{max} / cm^{-1} 3179 (H-N of maleimide), 1768 and 1694 (C=O of maleimide), 1548 (C=C of maleimide); HR-MS (MaXis) m/z found 208.0404, calc. 208.0403 ($[C_8H_{11}NO_2S+Na]^+$, 100%).

II. Fluorophore characterisation

Molecule	Solvent	ϕ_f %	ε_{max} $M^{-1}cm^{-1}$	$\lambda_{abs,max}$ nm	$\lambda_{ex,max}$ nm	$\lambda_{em,max}$ nm
**1**	Cyclohexane	28	5.8E+03	230, 407	232, 405	486
	Dioxane	10	4.9E+03	250, 402	262, 405	504
	Methanol	0.43	4.7E+03	251, 402	250, 402	546
	Water	Not soluble at 0.2 mM				
**2**	Cyclohexane	Not soluble at 0.2 mM				
	Dioxane	0.043	4.7E+03	247, 339	n/a	n/a
	Methanol	0.011	4.6E+03	244, 337	n/a	n/a
	Water	Not soluble at 0.2 mM				
**3**	Cyclohexane	31	3.7E+03	225, 357	235, 354	442
	Dioxane	38	4.5E+03	229, 367	233, 363	469
	Methanol	1.1	5.5E+03	229, 373	233, 370	514
	Water	Not soluble at 0.2 mM				
**4**	Cyclohexane	52	5.0E+03	231, 333	231, 334	412
	Dioxane	59	4.9E+03	233, 346	236, 346	450
	Methanol	2.8	5.5E+03	232, 357	233, 355	490
	Water	0.31	5.4E+03	231, 369	231, 365	520
**5**	Cyclohexane	0.16	7.3E+03	224, 380	232, 379	442
	Dioxane	0.15	6.6E+03	234, 386	233, 381	474
	Methanol	0.054	6.5E+03	236, 392	245, 396	515
	Water	0.037	5.7E+03	231, 410	232, 409	567
**6**	Cyclohexane	Not soluble at 0.2 mM				
	Dioxane	0.43	6.6E+03	238, 367	233, 364	460
	Methanol	0.20	6.6E+03	224, 374	233, 379	500
	Water	0.11	6.4E+03	217, 391	232, 393	535
**7**	Dioxane	35	5.0E+03	229, 364	233, 363	468
**8**	Dioxane	34	3.3E+03	231, 362	232, 364	466
**9**	Dioxane	0.052	6.7E+03	231, 375	n/a	n/a
**10**	Dioxane	0.017	9.9E+03	228, 240, 366	n/a	n/a
**11**	Dioxane	20	3.7E+03	240, 376	244, 374	486
**12**	Dioxane	0.94	3.7E+03	236, 378	241, 375	493
**13**	Dioxane	0.085	2.9E+03	262, 360	n/a	n/a
**14**	Dioxane	0.13	4.8E+03	245, 387	n/a	n/a

Table S1. Fluorescence spectroscopy data for compounds **1 - 14**.

III. Supplementary figures

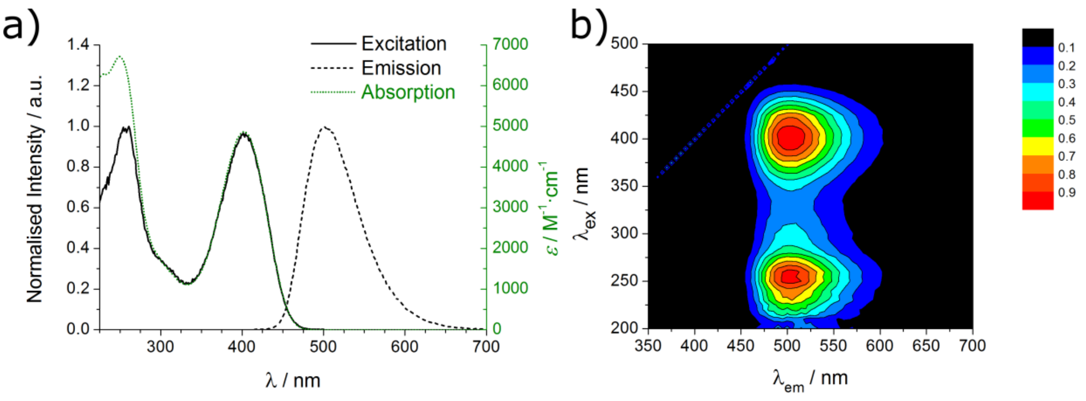

Fig. S1. a) Excitation, emission and absorption spectra of **1** in 1,4-dioxane at 10 µM; b) 2D excitation-emission spectra (with a 5 nm step) of **1** in 1,4-dioxane at 10 µM. Peaks at λ_{ex} = λ_{em} are due to Rayleigh scattering from the sample solution.

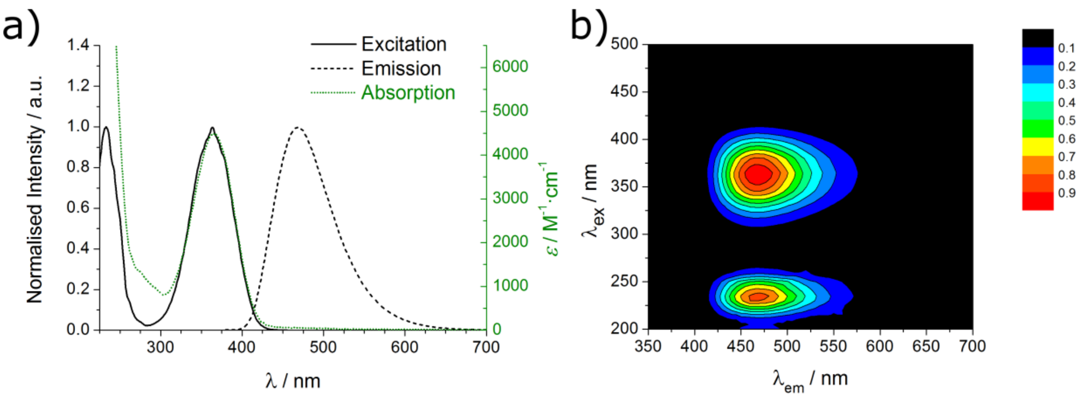

Fig. S2. a) Excitation, emission and absorption spectra of **3** in 1,4-dioxane at 10 µM; b) 2D excitation-emission spectra (with a 5 nm step) of **3** in 1,4-dioxane at 10 µM.

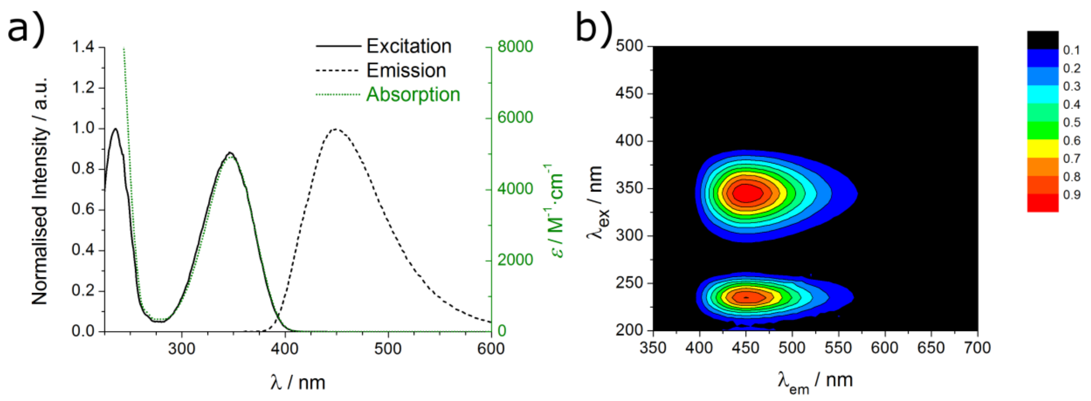

Fig. S3. a) Excitation, emission and absorption spectra of **4** in 1,4-dioxane at 10 μM; b) 2D excitation-emission spectra (with a 5 nm step) of **4** in 1,4-dioxane at 10 μM.

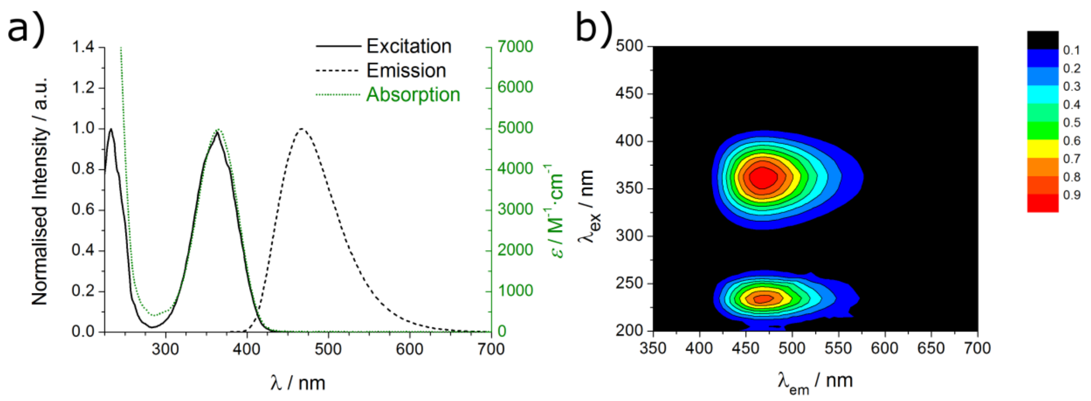

Fig. S4. a) Excitation, emission and absorption spectra of **7** in 1,4-dioxane at 10 μM; b) 2D excitation-emission spectra (with a 5 nm step) of **7** in 1,4-dioxane at 10 μM.

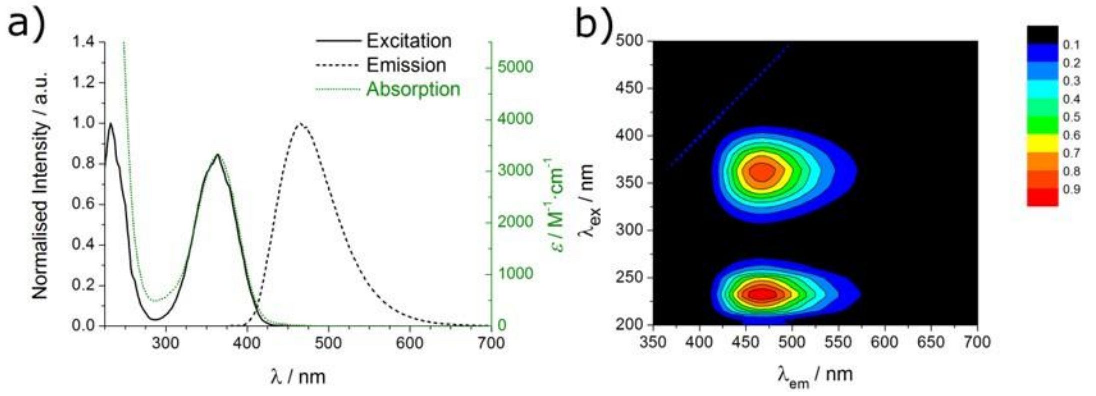

Fig. S5. a) Excitation, emission and absorption spectra of **8** in 1,4-dioxane at 10 μM; b) 2D excitation-emission spectra (with a 5 nm step) of **8** in 1,4-dioxane at 10 μM. Peaks at $\lambda_{ex} = \lambda_{em}$ are due to Rayleigh scattering from the sample solution.

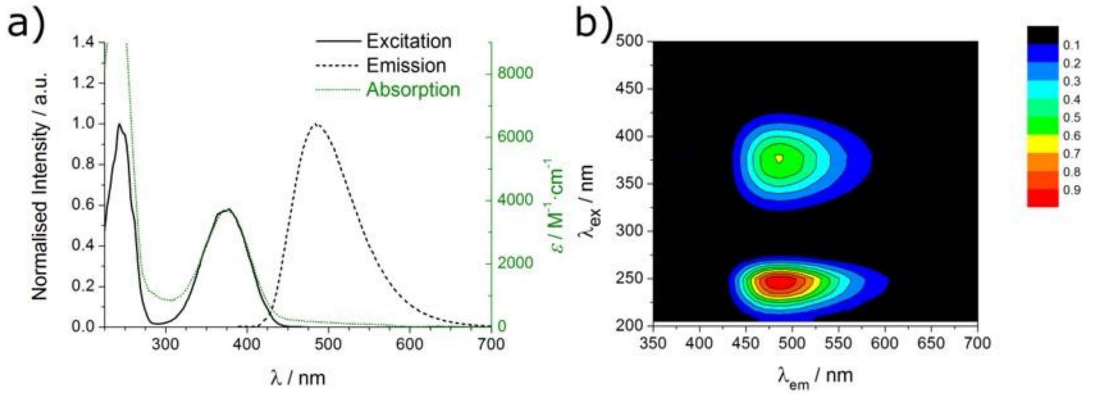

Fig. S6. a) Excitation, emission and absorption spectra of **11** in 1,4-dioxane at 10 μM; b) 2D excitation-emission spectra (with a 5 nm step) of **11** in 1,4-dioxane at 10 μM.

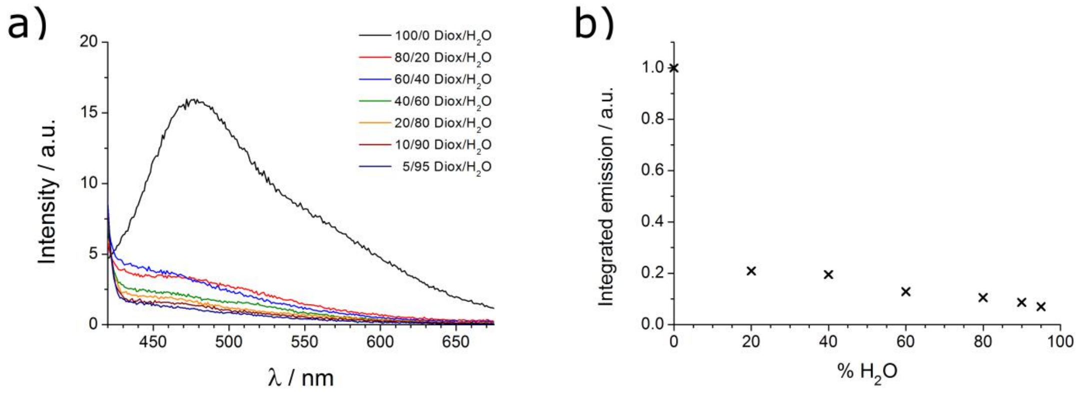

Fig. S7. a) Emission spectra of **9** (10 μM) in various ratios of 1,4-dioxane (Diox) and water; b) Plot of integrated emission against the water fraction.

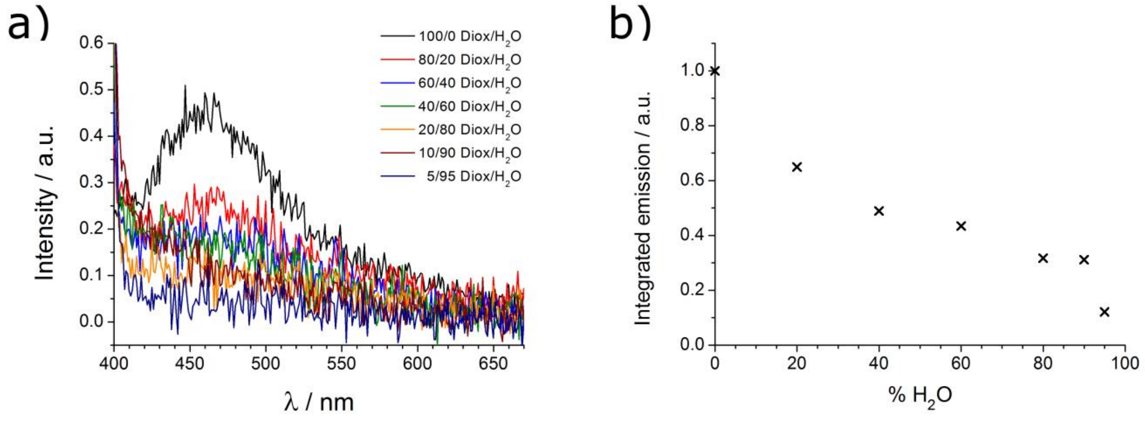

Fig. S8. a) Emission spectra of **10** (10 μM) in various ratios of 1,4-dioxane (Diox) and water; b) Plot of integrated emission against the water fraction.

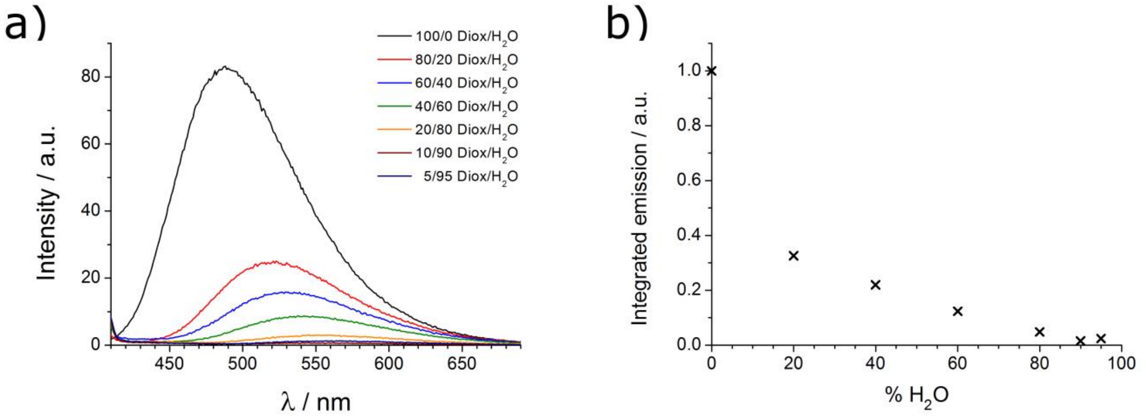

Fig. S9. a) Emission spectra of **12** (10 μM) in various ratios of 1,4-dioxane (Diox) and water; b) Plot of integrated emission against the water fraction.

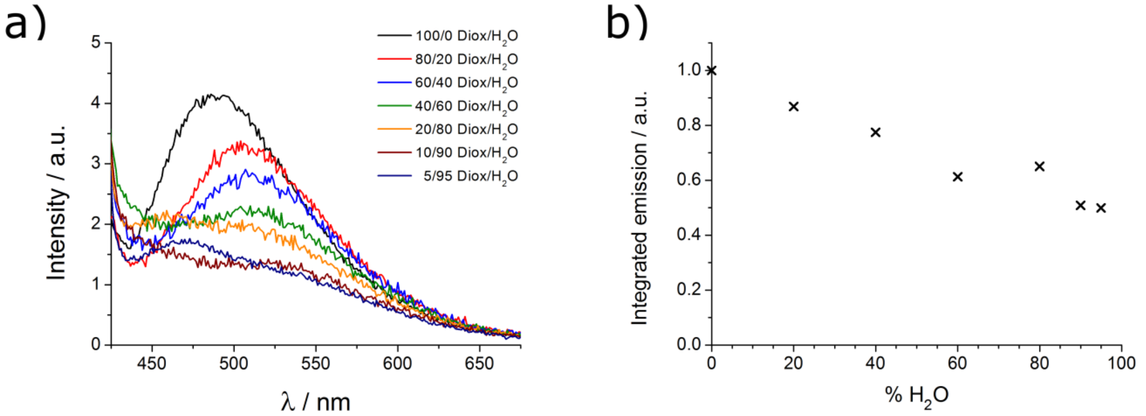

Fig. S10. a) Emission spectra of **13** (10 μM) in various ratios of 1,4-dioxane (Diox) and water; b) Plot of integrated emission against the water fraction.

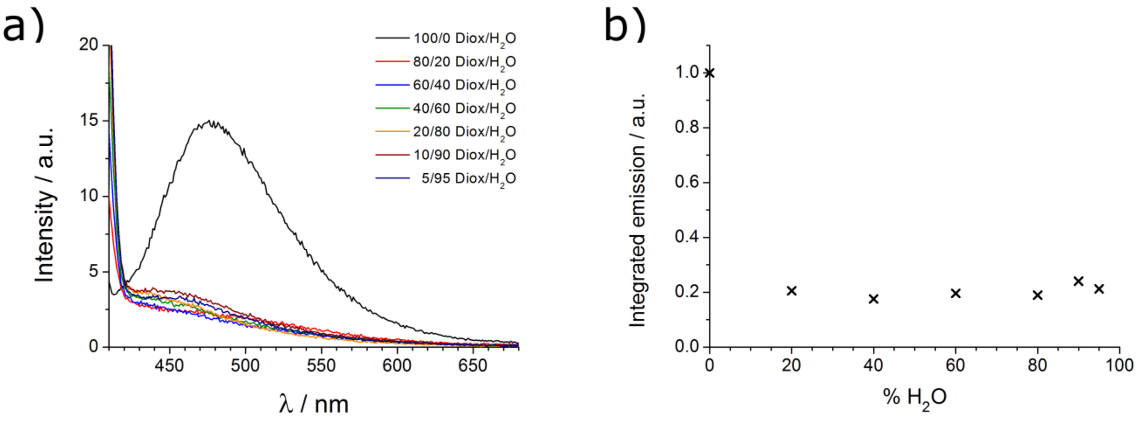

Fig. S11. a) Emission spectra of **14** (10 μM) in various ratios of 1,4-dioxane (Diox) and water; b) Plot of integrated emission against the water fraction.

IV. References

1. U. Muus, C. Hose, W. Yao, T. Kosakowska-Cholody, D. Farnsworth, M. Dyba, G. T. Lountos, D. S. Waugh, A. Monks, T. R. Burke, Jr and C. J. Michejda, *Bioorg. Med. Chem.*, 2010, **18**, 4535-4541.
2. R. Vanel, F. Berthiol, B. Bessières, C. Einhorn and J. Einhorn, *Synlett*, 2011, **22**, 1293-1295.
3. G. Martinez-Ariza, J. Dietrich, F. De Moliner and C. Hulme, *Synlett*, 2013, **24**, 1801-1804.
4. M. P. Robin, P. Wilson, A. B. Mabire, J. K. Kiviaho, J. E. Raymond, D. M. Haddleton and R. K. O'Reilly, *J. Am. Chem. Soc.*, 2013, **135**, 2875–2878.
5. E. Awuah and A. Capretta, *J. Org. Chem.*, 2011, **76**, 3122-3130.
6. J. T. Bowler, C. R. Clausen, D. J. Blackburn and W. Wu, *Tetrahedron Lett.*, 2014, **55**, 6465-6466.
